# Performance and Resource Requirements of In-Person, Voice Call, and Automated Telephone-Based Socioeconomic Data Collection Modalities for Community-Based Health Programs: A Systematic Review

**DOI:** 10.1001/jamanetworkopen.2022.43883

**Published:** 2022-11-28

**Authors:** Luke N. Allen, Shona Mackinnon, Iris Gordon, David Blane, Ana Patricia Marques, Stephen Gichuhi, Alice Mwangi, Matthew J. Burton, Nigel Bolster, David Macleod, Min Kim, Jacqueline Ramke, Andrew Bastawrous

**Affiliations:** 1 International Centre for Eye Health, Department of Clinical Research, London School of Hygiene & Tropical Medicine, London, United Kingdom; 2 NHS Education for Scotland, Glasgow, United Kingdom; 3 Institute of Health and Wellbeing, University of Glasgow, Glasgow, United Kingdom; 4 Department of Ophthalmology, University of Nairobi, Nairobi, Kenya; 5 Operation Eyesight, Nairobi, Kenya; 6 Peek Vision, Berkhamsted, United Kingdom; 7 London School of Hygiene & Tropical Medicine, London, United Kingdom; 8 International Statistics & Epidemiology Group, Department of Infectious Disease Epidemiology, London School of Hygiene & Tropical Medicine, London, United Kingdom; 9 School of Optometry and Vision Science, University of Auckland, Auckland, New Zealand

## Abstract

**Importance:**

Gathering data on socioeconomic status (SES) is a prerequisite for health programs that aim to improve equity. There is a lack of evidence on which approaches offer the best combination of reliability, cost, and acceptability.

**Objective:**

To compare the performance of different approaches to gathering data on SES in community health programs.

**Data Sources:**

A search of the Cochrane Library, MEDLINE, Embase, Global Health, ClinicalTrials.gov, the World Health Organization International Clinical Trials Registry Platform, and OpenGrey from 1999 to June 29, 2021, was conducted, with no language limits. Google Scholar was also searched and the reference lists of included articles were checked to identify further studies. The search was performed on June 29, 2021.

**Study Selection:**

Any empirical study design was eligible if it compared 2 or more modalities to elicit SES data from the following 3 categories: in-person, voice call, or automated telephone-based systems.

**Data Extraction and Synthesis:**

Two reviewers independently screened titles, abstracts, and full-text articles and extracted data. They also assessed the risk of bias using Cochrane tools and assessed the certainty of the evidence using the Grading of Recommendations, Assessment, Development and Evaluation approach. Findings were synthesized thematically without meta-analysis.

**Main Outcomes and Measures:**

Response rate, equivalence, time, costs, and acceptability to patients and health care professionals.

**Results:**

The searches returned 3943 records. The 11 included studies reported data on 14 036 individuals from 7 countries, collecting data on 11 socioeconomic domains using 2 or more of the following modes: in-person surveys, computer-assisted telephone interviews (CATIs), and 2 types of automated data collection: interactive voice response calls (IVRs) and web surveys. Response rates were greater than 80% for all modes except IVRs. Equivalence was high across all modes (Cohen κ > 0.5). There were insufficient data to make robust time and cost comparisons. Patients reported high levels of acceptability providing data via IVRs, web surveys, and CATIs.

**Conclusions and Relevance:**

Selecting an appropriate and cost-effective modality to elicit SES data is an important first step toward advancing equitable effective service coverage. This systematic review did not identify evidence that remote and automated data collection modes differed from human-led and in-person approaches in terms of reliability, cost, or acceptability.

## Key Points

**Question:**

What are the relative strengths and weaknesses of different socioeconomic data collection modes?

**Findings:**

In this systematic review of 11 studies with 14 036 individuals, high levels of equivalence and acceptability were found across in-person surveys, computer-assisted telephone interviews, and 2 types of automated data collection: interactive voice response calls and web surveys; cost and time comparisons were rarely performed. Response rates were greater than 80% for all modes except interactive voice response.

**Meaning:**

This systematic review identified no substantial evidence that remote and automated data collection modes are any worse than in-person approaches, and there was no compelling evidence that these approaches are faster or cost less.

## Teaser

This systematic review evaluates the use of interactive voice response calls and web surveys in obtaining socioeconomic data on individuals enrolled in community-based health programs.

## Introduction

### Rationale

Inequalities in health are pervasive and persistent. Women and girls, individuals living in rural areas, and persons with lower levels of income, education, and social status all tend to experience higher barriers to accessing care than other groups.^1,2,3,4^ To understand and redress socioeconomic inequalities, international development partners are increasingly calling for socioeconomic status (SES) data to be routinely collected and analyzed by all health systems and programs.^5,6^

Previous work has reported that SES data can be collected using a variety of modalities in the community setting, including in-person interviews, telephone calls, and automated telephone-based systems.^7^ There is growing interest in using mobile phones to collect data for global health programs on the basis that this modality is lower cost, faster, and more flexible than in-person approaches.^8,9^

Croke et al^10^ have argued that telephone-based data collection is acceptable in settings where mobile phone ownership rates exceed 80%. While this percentage is an arbitrary threshold, we note that the share of the population that has access to a telephone exceeds the proportion of those who own a telephone. Mobile phone ownership has increased sharply in the past decade such that there are now approximately 100 mobile phone subscriptions for every 100 people in low- and middle-income countries (LMICs).^11,12^ Across Sub-Saharan Africa, where telephone ownership is lowest, telephones have been used for a wide range of applications including surveillance, surveys, behavior change interventions, monitoring and evaluation, and training.^12,13,14,15,16,17^

It is well known that the mode of data collection (eg, in-person, telephone interview, or short message service [SMS]) can influence survey response rates and other performance characteristics, especially when the questions are of a sensitive nature.^18,19^ Previous research suggests that telephone-based data collection approaches may reduce social desirability bias—where responders provide what they perceive to be socially acceptable answers even if they are not accurate—compared with in-person approaches.^20^ However, telephone-based approaches also tend to have lower response rates and have historically presented under-coverage biases due to lower penetration among less-educated and low-income groups.^21^

Pariyo and colleagues^22^ have noted the dearth of research comparing different modalities of SES data collection in LMICs. Given the increasing feasibility and potential efficiency gains of using telephones for SES data collection, we aimed to systematically review the findings of empirical studies that have compared in-person vs voice call vs telephone-based modalities for gathering SES data for community-based health programs in terms of their performance characteristics, resource requirements, and acceptability to participants and service professionals.

## Methods

This registered review followed a published protocol.^23^ It also followed the Preferred Reporting Items for Systematic Reviews and Meta-analyses (PRISMA) reporting guideline and Cochrane guidelines.^24,25^

### Eligibility Criteria

#### Population

In this systematic review, the population was composed of studies rather than people, namely, those that sought to compare 2 or more modalities for SES data collection from individuals enrolled in community-based health programs. Studies that reported on only 1 mode of data collection were excluded.

For the purpose of this review, *health programs* were defined as organized activities to improve 1 or more health outcomes in a defined population. *Community-based* encompasses all settings except hospitals. Some researchers exclude primary care facilities from definitions of community-based care^26^; however, these facilities were included in this review, along with outreach and mobile clinics, community centers, schools, workplaces, and people’s own homes.

*Socioeconomic status* is a critically important but nebulous concept that pertains to social and economic standing within society.^27^ It determines exposure to the social determinants of health; “the conditions in which people are born, grow, live, work, and age”^2^; and relates to issues of privilege, power, and control.^28^ Almost all health outcomes are patterned according to SES, with the most disadvantaged populations experiencing the worst health outcomes.^2,28,29^ Socioeconomic status is commonly measured using income, educational level, occupation, and other metrics, such as wealth, caste, and place of residence. We included all of these domains, as well as any other proxies that are identified by researchers as capturing SES.

#### Interventions

The interventions being studied are 3 different groups of modalities for collecting SES data (Box). The focus is on the modality of data collection (eg, in-person vs voice call vs automated) rather than the content of the wording that is used to elicit information.

Box. **Definitions of the 3 Data Collection Approaches Used in This Review**

**In-person data collection** included any form of exchange between a program implementer and a participant or their responsible guardian where the program implementer asks predefined questions to ascertain the participants’ socioeconomic status and a synchronous response is received, ie, both parties occupy the same time and space, and the response is recorded by the implementer before the encounter is terminated. Any recording modality used by the program implementer will be included, such as pen and paper or completion of an electronic form. For this review we will also include self-administered questionnaires as a subtype of in-person data collection, provided that the data collection instrument was provided when the participant presented to a program implementer in person, the participant was asked to complete the data entry form, and the participant submitted their responses before departing. Any nonhospital location was accepted.
**Voice call data collection** includes real-time, telephone-based verbal exchanges between program implementers and participants whereby SES data are elicited and recorded by the program implementer using predefined questions. This category included computer-assisted telephone interviews—where the interviewer follows prompts on a computer screen, usually in a call center—as well as non–computer-assisted telephone interviews. Videocalls were included as another subtype of voice calls.
**Automated telephone-based data collection** included any mobile telephone–based asynchronous exchange of information whereby participants are sent a standardized text message (also known as a short message service [SMS]), multimedia message (MMS), or automated phone call (sometimes called interactive voice response or IVR) and asked to provide SES data. Interactive voice response calls use prerecorded messages that prompt respondents to provide answers using speech, eg, state your age in years or by entering numbers on the keypad eg, press 1 for yes and 2 for no. We allowed responses to be provided using the same modality or any other digital form, eg, entering details on a webpage/web survey. Interventions that required participants to engage with human program implementers (eg, human-led SMS exchanges) were excluded from this modality. All forms of phrasing of the requests and responses were included. Reasoning that all smartphones come with a preloaded browser, we included web surveys that can be accessed by a hyperlink, as long as the link was sent via SMS or MMS. We excluded data collection approaches that required the download of third-party software, including email.

We excluded approaches that used a blend of modes to elicit SES data. We excluded studies in which the SES questions and wording were not kept constant across modes. Studies that gather SES data at the household or community level were only included if these data were used to make assumptions about the SES of identifiable individual participants enrolled or due to be enrolled in the service delivery program of interest.

#### Comparator

Studies that examined any 2 or more modalities were eligible. We excluded studies that only reported outcomes for 1 modality, that is, in which comparisons were not possible between modes. There was no index or gold standard data collection modality. Interventions that bundled requests for SES data with requests for other data (eg, broader demographic data) were included, as long as separate results were reported for the SES data collection element.

### Outcomes

Our 2 primary outcomes were performance characteristics and resource requirements. We reported these outcomes at the level of the following individual SES items.

#### Performance Characteristics

- Response rate: number of completed SES items divided by the total number of elicitation attempts. This outcome was calculated at the level of each SES item.
- Equivalence: agreement between the responses obtained from 2 or more different modalities. Recognizing that equivalence can vary by question, we report equivalence for each SES item. We report equivalence figures that aggregated multiple SES questions in a secondary analysis; however, we do not report aggregate equivalence figures that mixed SES items with non-SES items. Following Marcano Belisario et al^30^ and Gwaltney et al,^31^ we used comparisons of mean scores between modalities and/or correlations and/or measures of agreement, including intraclass correlation coefficients, Pearson product-moment correlations, Spearman ρ, and weighted κ coefficients.

#### Resource Requirements

- Time: the time taken to gather SES data using each approach (range and mean).
- Costs: any financial data on the costs of operating the data collection approach. These approaches include fixed costs (equipment, software, insurance, and personnel required to set up a given data elicitation modality) and ongoing support costs. We aimed to calculate the fixed and per-person costs to purchasers per completed survey.

Our secondary outcome was acceptability to participants and service professionals, based on survey or interview results reporting on how program implementers and participants perceived the collection modality in terms of intrusiveness, ease of use, time requirement, and general acceptability, as well as perceived advantages, barriers, disadvantages, and additional costs presented by the beneficiaries, data collectors, or study authors.

#### Measures of Effect

For each outcome we present raw values and risks ratios. We used the most commonly studied modality (computer-assisted telephone interview [CATI]) as the reference group.

### Study Types to Be Included

All empirical study designs that compared 2 or more data collection modalities were included. Studies were only included if they compared modalities that had been used to gather data from participants. Studies that used simulated data or data obtained from populations other than the intended beneficiaries were excluded. Both quantitative and qualitative study designs were included as long as they reported 1 or more of the outcomes of interest. Review articles were excluded, but the primary studies they discussed were screened for potential inclusion.

### Information Sources

We searched the following information resources: the Cochrane Library, MEDLINE, Embase, Global Health, ClinicalTrials.gov, and the World Health Organization International Clinical Trials Registry Platform for current and ongoing trials. We searched OpenGrey for gray literature and the first 20 pages of Google Scholar. We checked the reference lists of included studies and relevant systematic reviews to identify any additional potentially relevant reports of studies. We contacted key authors to uncover additional or upcoming studies.

### Search Strategy

The search strategy was built around 3 blocks: data collection modalities, SES concepts, and study design and setting terms (eMethods in the Supplement provides the full strategy). The search was limited to human studies published since 1999 (the year that it first became possible to send cross-network SMS messages). We searched for full-text studies published in any language. We did not include reports of studies published as conference abstracts. The search was performed on June 29, 2021.

### Study Selection

Two of us (L.N.A. and S.M.) independently screened all titles and abstracts and full texts using online software (Covidence). Studies that did not meet the inclusion criteria were excluded. Disagreements were resolved through consensus-based discussion and discussion with a third reviewer (D.B.) when necessary. We recorded reasons for exclusion at the full-text screening stage.

### Data Extraction and Management

Two of us (L.N.A. and S.M.) independently extracted study characteristics and data from the included studies using a custom data extraction form that was based on the Cochrane template.^25^ We emailed study authors to request additional information and primary data if any aspect of their article precluded the assessment of eligibility or inclusion in the data synthesis.

#### Data Items

We extracted the following items from each study:

- Article details
- Study design, population, and setting
- Questions used to assess SES (SES domains and individual response options)
- Number of times SES data were collected from each participant (eg, cross-sectional or serial)
- Modalities used to collect SES data:
  - Modality name and definition
  - Who gathered the SES data
  - When data were gathered in the patient journey/program
  - Equipment used
  - Who provided the data
  - Synchronous or asynchronous data collection
- Types of comparison and outcome measures
- Outcomes: response rate, completeness, equivalence, time, costs, and all qualitative text provided on acceptability

### Risk of Bias Assessment for Included Studies

Two of us (L.N.A. and S.M.) independently assessed risk of bias using the Cochrane RoB2 tool for randomized studies^32^ and ROBINS-I^33^ for nonrandomized studies. Disagreements were resolved by consensus and discussion with a third reviewer (D.B.) if necessary. The risk of bias for each outcome across individual studies was summarized by risk of bias tables. We also produced a review-level narrative summary of the risk of bias.

### Principal Summary Measures

We used ratios to present principal differences between modalities as we considered the relative level of agreement, cost, or acceptability between each approach for a given SES item to be more important than the absolute level.

### Strategy for Data Synthesis

Had data been available, we planned to pool effect estimates using a random-effects model.^34^ Given the heterogeneity in study design, interventions, and outcomes of the included studies, we used a narrative synthesis without a meta-analysis approach, following reporting guidelines from Campbell and colleagues.^35^ We stratified the synthesis by modality, SES domain, and outcome. We assessed heterogeneity by considering study design, interventions, and outcomes. To assess the risk of bias across studies we assessed selective outcome reporting by comparing protocols (when available) with published reports.

### Additional Analyses

We planned to exclude studies at high risk of bias from the synthesis and primary analysis. We planned to perform a secondary analysis that included all studies irrespective of their risk of bias. We also planned to perform a secondary analysis assessing whether findings differed between high-income and LMICs.

### Assessment of Certainty of Evidence

We used the Grading of Recommendations, Assessment, Development and Evaluations criteria to assess the certainty of the primary outcomes.^36,37^ One of us (L.N.A.) collated the evidence for each primary outcome and suggested initial ratings that were discussed with another of us (S.M.) and agreed on by joint decision. For randomized clinical trials, evidence was assumed to be of high certainty and then downgraded due to risk of bias, inconsistency of results, indirectness of evidence, imprecision, or publication bias. For observational studies, evidence started at low certainty but was upgraded for a large effect size, dose-response, gradient, or plausible confounding that decreases the magnitude of effect.

## Results

### Search Results

**Figure 1.**
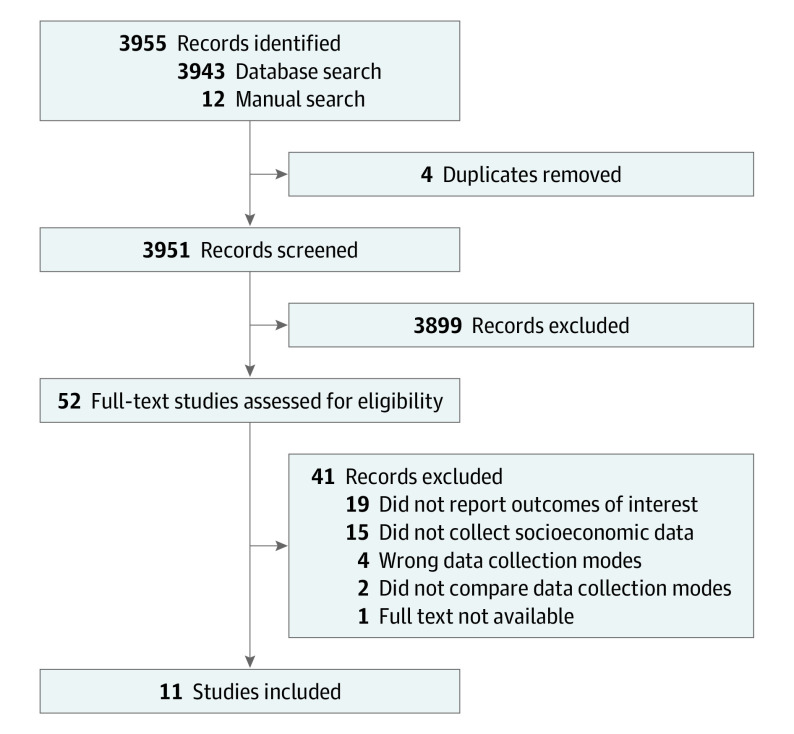
Study Flow Diagram

Our search returned 3943 records and additional searches returned a further 11 studies (Figure 1). We contacted 24 study authors for full texts or missing data. Only 1 study^38^ was excluded because we could not obtain the full text.

### Study Characteristics

**Figure 2.**
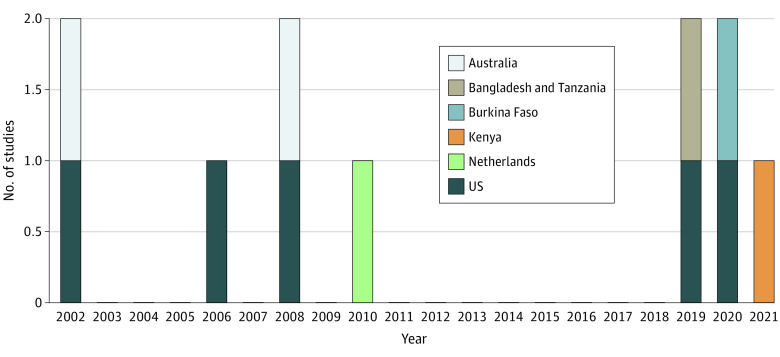
Publication Year and Study Population Location of Included Studies

The 11 included studies reported data on 14 036 individuals from 7 countries: 5 from the US,^39,40,41,42,43^ 2 from Australia,^44,45^ and 1 each from Bangladesh and Tanzania,^22^ Burkina Faso,^46^ Kenya,^47^ and the Netherlands^48^ (Figure 2). As such, 3 studies (27.3%) reported data from 4 LMICs. All studies were published in English. Table 1 summarizes the included studies’ designs, modes used, SES domains, and outcomes.

**Table 1.** Study Characteristics of Included Studies Reporting the Performance Characteristic of 2 or More SES Data Collection Modes

Source	Design	Population	Study focus	Modes used to collect SES data	SES domains	Outcome domains
Corkrey and Parkinson,^44^ 2002, Australia	Parallel, randomized, 4-arm survey	2880 Adults with fixed telephone connections, nationally representative sample	Drugs and alcohol use survey	CATI, IVR, hybrid CATI/IVR	Educational level, marital status, country of birth, employment	Costs, acceptability
Ellen et al,^39^ 2002, US	Randomized, parallel 2-arm survey	223 African American adolescents living in San Francisco	Teen sexual behavior data collection	CATI, web survey	Household structure, school enrollment	Costs, acceptability
Graham et al,^43^ 2006, US	Test-retest: CATI followed by web survey 2 d later	213 Internet users who searched for stop smoking and navigated to the intervention site	Smoking habits survey, nested within a RCT testing a smoking cessation intervention	CATI, web survey	Household income	Equivalence (Cohen κ)
Graham and Papandonatos,^42^ 2008, US	Test-retest: CATI then web 2 d later	422 Internet users who searched for stop smoking and navigated to the intervention site	Smoking habits survey, nested within an RCT testing a smoking cessation intervention	CATI, web survey	Household income	Equivalence (Cohen κ)
Chittleborough et al,^45^ 2008 Australia	Test-retest: in-person then CATI 6 mo later	2206 South Australian adults living in metropolitan areas and listed in the electronic white pages	SES data collection	CATI, in-person	Parental educational level, occupation, employment status, household income, educational level, urban/rural, country of birth, marital status	Response rate
Nagelhout et al,^48^ 2010, the Netherlands	Randomized, parallel, 2-arm survey	2072 Adult smokers registered with an online survey database	Tobacco use data collection	CATI, web survey	Educational level, marital status	Response rate, time, costs
English et al,^40^ 2019, US	Parallel, randomized, 2-arm survey	900 Adults from rural American Indian communities in New Mexico	General public health survey	CATI, in-person	Educational level; household income, employment status	Response rate, time, costs
Pariyo et al,^22^ 2019, Bangladesh and Tanzania	Randomized crossover survey	2196 Adults with mobile phone access in Bangladesh and Tanzania	Noncommunicable diseases data collection	CATI, IVR	Education, urban/rural	Equivalence (Cohen κ)
Gagliardi et al,^41^ 2020, US	Parallel, nonrandomized, 2-arm study	1008 Women overdue for cancer screening in a US health system	Primary care cancer screening outreach	CATI, IVR	Primary care registration	Costs
Greenleaf et al,^46^ 2020, Burkina Faso	Randomized, parallel 2-arm survey	1766 Women aged 15-49 y who own a mobile phone	Family planning data collection	CATI, IVR^a^	Educational level, marital status, urban/rural	Response rate, equivalence (Cohen κ), costs, time
Ashigbie et al,^47^ 2021, Kenya	Test-retest CATI then in person <24 h later for a 10% subsample	130 Adults registered with Kenyan health facilities	Access to medicines survey	CATI, in person	Educational level, wealth	Time, costs

Abbreviations: CATI, computer-assisted telephone interview; IVR, interactive voice response; RCT, randomized clinical trial; SES, socioeconomic status.

^a^ Greenleaf et al^46^ used hybrid-IVR: participants were first called by a researcher to set up the process and take consent, and the participant was then transferred to an IVR system for data collection.

#### Study Designs

One study used a randomized crossover survey design.^22^ Parallel 2-arm^39,40,48^ and 4-arm^44^ surveys were more prevalent, with participants randomly allocated to different survey instruments and comparisons made between the instruments. Gagliardi et al^41^ used a nonrandomized parallel 2-arm approach. Greenleaf et al^46^ randomized participants to CATIs or interactive voice response calls (IVRs) and compared response rates between arms, but also compared both arms with findings from an in-person survey completed 11 months previously to calculate equivalence. The 4 remaining studies used test-retest approaches.^42,43,45,47^ The vast majority of studies collected SES data as part of broader surveys. Only Chittleborough et al^45^ had the primary aim of comparing different modalities for collecting SES data.

### Risk of Bias

eFigure 1 and eFigure 2 in the Supplement summarize the risk of bias for each study. Overall, 7 studies were found to be at low risk of bias; we had some concerns regarding 4 studies, and none were found to be at high risk of bias. The risk of bias across studies (including selective outcome reporting) was low to moderate.

### Data Collection Modalities

None of the included studies used SMS, multimedia message (MMS), or non-CATI approaches. CATIs were used in all 11 studies: this approach entails conducting real-time telephone calls and leading participants though a series of questions read from a computer screen, with responses usually entered using the same program. Three studies used data collected as part of an existing national survey,^44,45,48^ 1 study used data collected by primary care administrative staff as part of an implementation and comparative effectiveness study,^41^ and the remaining 7 studies used members of the research team to collect the data; in 3 of these studies data were collected as part of a larger parent study.^42,43,47^

In-person data collection,^40,45,46,47^ web surveys,^39,42,43,48^ and IVRs^22,41,44,46^ were each assessed by 4 studies. Two studies^44,46^ included hybrid IVR arms when a researcher called the participant at the beginning or end of the IVR data collection activity. We included these studies because all SES data were collected during the IVR phase; however, we have singled these studies out in the ensuing analyses because we might expect this approach to achieve a higher response rate than IVR approaches with no associated human interaction. All of the studies directly compared CATIs against one other approach except for Greenleaf et al,^46^ who compared CATIs against IVRs for response rate, time, and costs, and they compared CATI and IVR approaches against in-person survey for equivalence. eFigure 3 in the Supplement illustrates the comparisons made between each modality.

### Socioeconomic Domains

Eleven different SES domains were reported across the 11 included studies (eTable 1 in the Supplement). More than one-third of the studies collected data on educational level, marital status, household income, and employment; however, multiple different response options were used, and no 2 studies used exactly the same wording or response options. eTable 2 in the Supplement provides the survey items and response options used for each SES domain within each study.

### Response Rate

Four studies presented data on the response rates for individual questions, defined as the number of completed SES responses divided by the total number of elicitation attempts (Table 2). Not every study provided sufficient data to permit the calculation of 95% CIs.

**Table 2.** Response Rates

Source	Domains^a^	CATI response rate, %	Response rate, comparator, %	Ratio CATI/comparator
Chittleborough et al,^45^ 2008	Highest level of education	100	100 In person	1.00
	Occupation (6 categories)	100	100 In person	1.00
	Employment status (7 categories)	99	98.1 In person	1.01
	Household income (4 categories)	89.2	88.4 In person	1.01
	Area of residence (metropolitan/country)	100	100 In person	1.00
	Marital status	100	100 In person	1.00
	Country of birth	100	100 In person	1.00
Pariyo et al,^22^ 2019	Residential area	100	68 IVR	1.47
	Ever attended school	100	71 IVR	1.41
	Marital status	100	73 IVR	1.37
Nagelhout et al,^48^ 2010	Educational level	96.8	99.2 Web survey	0.98
	Marital status	99.5	99.7 Web survey	1.00
Gagliardi et al,^41^ 2020	Insurance	99.3	99.5 IVR	1.00

Abbreviations: CATI, computer-assisted telephone interview; IVR, interactive voice response.

^a^ The denominator for each domain is the entire population for each study listed in Table 1.

Socioeconomic status data collection using CATIs was found to have either superior or equivalent response rates compared with IVRs. The response rates were found to be similarly high in each domain by Gagliardi et al^41^ and 100% in all the SES domains collected by Greenleaf et al,^9^ whereas response rates using IVRs ranged from 68% to 73%. Nagelhout et al^48^ found response rates using CATIs and web-based data collection to be similarly high.

Chittleborough et al,^45^ the only study to report response rates for individual SES domain-level questions, compared CATIs and in-person data collection and found similar response rates between the 2 methods, although English et al^40^ reported an overall survey completion rate of 35.7% using CATIs compared with 68.9% in-person—a ratio of 0.52. English et al^40^ also reported that this lower rate was noted despite the fact that the CATI was significantly shorter (25 vs 45 minutes). A potential confounding factor was that a nominal incentive was offered to individuals who completed the in-person survey, but this was not logistically possible to offer those completing CATIs, although the English et al^40^ highlighted that the interviewers were trained not to mention the incentive until after the survey had been completed to reduce the risk of bias.

### Equivalence

Six studies assessed the level of agreement between the SES responses obtained from 2 or more different modalities. All used weighted κ coefficients. eTable 3 in the Supplement presents findings by SES domain. In a crossover design, Pariyo et al^22^ presented 2 sets of coefficients for each indicator depending on which modality was used first. The authors provided no interpretation for the very low agreement between IVRs and CATIs for education in Tanzania. They noted that the higher levels of agreement observed with performing IVRs first for other domains (which extend beyond the 2 SES domains presented herein) may be due to a form of selection bias where less-educated people may drop out of IVRs.^22^ Apart from this domain, all other κ values were greater than 0.51, which Cohen^49^ suggested interpreting as moderate agreement, with many exceeding 0.8: almost perfect agreement.

### Time

Three studies quantified the time taken to gather SES data using different approaches (eTable 4 in the Supplement). None presented ranges and Nagelhout et al^48^ and English et al^40^ did not present times for both of the approaches that they used. All 3 studies presented the time taken to complete the entire survey—not just the SES instruments. Ellen et al^39^ and Nagelhout et al^48^ used the same number and wording of questions irrespective of modality. Ashigbie et al^47^ found that CATIs were 1.48 times slower than in-person surveying, but crucially, this did not include the time taken to travel to each household.

### Costs

Seven studies presented cost data^39,40,41,44,46,47,48^; however, there was little consistency in the cost items included in the estimations for each modality and, in some cases, specific details of costs included were not provided. All studies that reported cost data compared CATIs with another mode of data collation, and there was notable variability in the cost-effectiveness, measured as cost per completed interview, of the different modalities between the studies related to response rates, interviewer costs, and participant reimbursement. We present the ratio of CATIs to other modes in eTable 5 in the Supplement.

Two studies compared CATIs with in-person interviewing: English et al^40^ found that both methods incurred high costs, but in-person interviewing was more cost-effective than telephone per completed survey due to the low response rate of telephone administration among American Indian or Alaska Native rural populations. Conversely, Ashigbie et al^47^ found telephone interviewing to be less expensive than in-person interviewing in semiurban and rural communities in Kenya. Although the interviews took longer, the process was less time-consuming because data collectors did not have to travel, often via poor road networks, to houses that may not be close to each other, incurring further cost. Nagelhout et al^48^ found web surveys to be more cost-effective than CATIs due to lower fieldwork costs and slightly lower participant reimbursements required, while Ellen et al^39^ found web surveys to be more expensive when combining actual costs for interviewers, mailing, telephones, travel, incentives, and supplies.

One study found IVRs to be more cost-effective than CATIs owing to reduced personnel costs,^41^ but 2 studies^44,46^ found IVRs to be less cost-effective due to the costs associated with recording the automated survey in multiple languages, additional airtime costs to complete the survey, and lower completion rates.

### Acceptability

None of the studies explored acceptability to providers. Two studies presented data on acceptability to participants: Ellen et al^39^ found no statistically significant differences (*P* > .05) in perceived comfort, honesty, and accuracy in answering full surveys delivered by CATIs vs web survey. We note that Ellen et al^39^ did not single out acceptability of the SES-specific questions. Corkrey and Parkinson^44^ assessed participants’ perception of ease, enjoyment, stress, and likability using IVRs and CATIs. Both methods scored equally highly for all 4 domains. eTable 6 in the Supplement presents the GRADE level of certainty for each of the key findings from the review’s primary outcomes.

### Secondary Analyses

None of the studies had high risk of bias, so none were excluded from the primary analyses. When we repeated the analyses comparing studies conducted in high-income vs LMIC settings we found that there were insufficient data to compare equivalence or time requirements for different modes. Greenleaf et al^46^ found a lower response rate with IVRs in Burkina Faso (72%) than Nagelhout et al^48^ found with the same modality in the Netherlands (99%); however, participants in the latter study were financially reimbursed, so this example is not a fair comparison. Ashigbie et al^47^ and Greenleaf et al^46^ both obtained very high CATI response rates (>95%) in LMICs; however, response rates were similarly high for the same items asked in high-income settings.

The cost per completed CATIs ranged from AU $6 to US $211 (approximately AU $7 and US $240 in 2022) depending on accounting practices. Heterogeneity in the application of each method and accounting practices precludes any firm conclusions, but data collection modes used in LMICs do not appear to be systematically more or less expensive than those used in high-income countries.

## Discussion

### Summary of Main Findings

Our systematic review included 11 studies that collected data on 11 different SES domains using 4 different modalities under the 3 overarching categories of in-person, voice call, and automated approaches. All studies used CATIs, 4 used web surveys, 4 used in-person approaches, and 3 studies used IVR methods. None of the included studies used SMS data collection, and all of the in-person approaches involved home visits. Despite an overall low risk of bias across the studies, comparisons were limited by marked heterogeneity in the SES items used.

There is not enough evidence to say whether automated approaches are less costly than nonautomated data collection modalities. This lack of evidence is mainly due to differences in costing approaches used, as well as heterogeneity in how each modality was used. Only Ashigbie et al^47^ compared the time taken to complete surveys, finding that CATI was 1.48 times slower than in-person elicitation; however, their figure did not include the travel time involved for home visits so the level of certainty for this finding is very low. Two studies compared the acceptability of CATIs vs IVR^44^ and CATIs vs web survey,^39^ finding no statistically significant differences in reported comfort, honesty, accuracy, ease, enjoyment, stress, or likability, which were assessed at the level of the whole survey rather than isolating the SES questions.

We can be moderately certain that response rate is equally high for SES questions asked via CATI, web survey, and in-person interview. Response rates may be slightly lower for IVR than for other modes, which may be largely related to incomplete responses. Greenleaf et al^46^ found high rates of break-off, where 19.7% of individuals (n = 174) consented but answered less than 50% of the relevant questions using this method. We postulate that human-led interactions exert a stronger social pressure not to terminate the call partway through the interview.

Equivalence between answers elicited using automated vs nonautomated approaches was moderate to substantial for all comparisons made. Responses provided by CATIs seem to be equivalent to those provided by web survey and in-person interviews.

Equivalence was also generally moderate to high between CATI and IVR, with the marked exception of eliciting educational attainment in Tanzania (κ = 0.03), where there appeared to be systematic underreporting at initial IVR compared with CATI follow-up. This finding suggests that there may have been a systematic issue in understanding this prerecorded question. The authors also noted that if a respondent accidentally entered an incorrect option on IVR, there was no facility to change their answer.^22^

In sum, CATI, web surveys, and in-person approaches can all attain very high response rates and appear to collect equivalent data. Our review found a slightly lower response rate with IVRs than the other modes, although this finding is based on 2 studies. We did not find sufficient evidence to suggest that time requirements, costs, or acceptability vary meaningfully between modes. Automated approaches (ie, web surveys and IVRs) have comparable response rates and similarly high perceived levels of acceptability compared with surveys conducted in person or with the telephone, although there are very few studies contributing evidence.

The time and costs for each mode seem to depend on the baseline telephone response rate for the population of interest and the distances involved in home visits: sometimes it may be more cost-effective to visit households than to repeatedly call. The length of telephone calls can also be a material factor when airtime is expensive, and there is low-quality evidence to suggest that IVRs may take longer than human-led calls. However, we note that IVRs do not involve personnel costs beyond setting up and managing the software.

### Comparisons With the Wider Literature

The World Health Organization recommends that health programs and researchers should routinely gather socioeconomic data on a wide range of domains.^5,6^ We note that none of our included studies collected data on religion, sexuality, or disability.

We found that respondents using IVRs and CATIs felt they were honest with their answers, even when answering sensitive questions. The wider evidence suggests that automated approaches, such as IVRs and web surveys, may obtain more honest answers than CATIs or in-person interviews^15,50,51,52^ due to reduced social distance and desirability bias.^22^ Automated approaches may also reduce bias that can arise from the social dynamics of interacting with a human, such as acquiescence^18,53^ and nonuniform questions, because a computer presents the same question in the same way every time, whereas a person does not.^54^ Social dynamics involved in providing answers to a real person may reduce the risk of satisficing (ie, providing the first/easiest option to complete the survey quickly).^55^ We did not find evidence to support or refute this hypothesis. Self-administered approaches, such as web surveys, may place a higher cognitive burden on respondents that can lead to disengagement^53^ and satisficing.^55^ Coupled with our findings that web surveys tended to achieve low response rates and were not much less costly than other options, we recommend that researchers consider using alternative options. One final important source of difference between automated and nonautomated modes is the measurement error that can stem from the fact that respondents can ask for clarifications and amend their answers, whereas these options are often not available for IVR and some web survey modes.^22^

We did not find enough data to make robust comparisons between the use of different modes in LMICs vs high-income countries. Reviews conducted by Gibson et al^12^ and Greenleaf et al^9^ suggest that more research is required to understand the reliability and accuracy of different modes in low-income settings.

In 2015, Ballivian and colleagues^8^ argued that telephone-based data collection approaches can introduce selection bias. This argument is less of a problem now that telephone ownership is so high around the world; however, low-income groups may be the least likely to own mobile phones and this is a material consideration for work seeking to obtain representative SES data for a given population. Remote and rural communities may also have unreliable network coverage. A further issue raised by Ballivian and colleagues^8^ is the lower response rates from telephone-based approaches vs face-to-face data collection modes; however, we did not find this factor to be an issue in the included studies.

None of our included studies examined SMS/MMS or clinic-based data collection. A 2008 study from a California ambulatory care service found that collecting race and ethnicity and language data using a paper questionnaire at the front desk yielded an 88% response rate at a cost of $0.21 per completed survey.^56^ West and colleagues^57^ found that CATIs were faster and less costly than manual SMS data collection for a 15-item survey of Nepalese adults. These studies were excluded from our review because they did not use comparators.

### Strengths and Limitations

Our study had a number of strengths: our search was designed by a Cochrane information specialist (I.G.), and we included a wide range of databases and other sources. We used independent dual screening, data extraction, and quality scoring, and followed best practice guidelines throughout the study. We included a wide range of outcomes to maximize the utility of the review for program managers faced with difficult decisions about which modality to use.

This study has limitations. The performance of individual SES items in a given questionnaire is likely to be influenced by the preceding items, the focus of the overall survey, and broader contextual factors. To minimize bias, we calculated and reported intermodal comparison rates rather than reporting absolute levels. Although this approach is methodologically robust, decision-makers are unlikely to select a mode on the basis of how it performs for individual survey items. We did not search for or extract data on sample frame errors and nonresponse errors.^58^ We excluded articles that were published before 1999, which may have excluded useful studies. We note that not all telephones can be used to access web surveys.

## Conclusions

Our review reinforces the message that the choice of survey mode should be guided by the type of questions being asked, the population, and the resources available.^8,10^ We found that CATIs, IVRs, web surveys, and in-person interviews have all been used to attain high response rates with comparable answers in a range of settings. Marked heterogeneity in their deployment makes it very difficult to reach conclusions about their relative costs and benefits, and future work should aim to align accounting practices with those used by major reviews. Given the absence of evidence that automated and telephone-based systems deliver inferior data, we recommend that decision-makers try approaches that are likely to offer cost savings; however, it is important to review response rates early on and consider the extent to which selection bias is influencing the findings.

## Notes

Supplement. **eMethods.** Search Strategy **eFigure 1.** RoB-2 Risk of Bias Table for Randomized Studies **eFigure 2.** ROBINS-I Risk of Bias Table for Nonrandomized Studies **eFigure 3.** Comparisons Between Data Collection Modes **eTable 1.** Socioeconomic Domains Used in the Included Studies **eTable 2.** Response Options Within Each SES Domain **eTable 3.** Equivalence Between Modalities of 6 Studies Reporting Equivalence Between Data Collection Modes **eTable 4.** Time Requirement for Each Modality **eTable 5.** Cost per Completed Interview for Different Modes **eTable 6.** Summary of Key Findings
eReferences
