# Supplementary material for: Performance and Resource Requirements of In-Person, Voice Call, and Automated Telephone-Based Socioeconomic Data Collection Modalities for Community-Based Health Programs: A Systematic Review
Source: JAMA Netw Open. 2022 Nov 28;5(11):e2243883. doi: 10.1001/jamanetworkopen.2022.43883 (PMC9706363; doi:10.1001/jamanetworkopen.2022.43883)
Supplement: Supplement. — eMethods. Search Strategy eFigure 1. RoB-2 Risk of Bias Table for Randomized Studies eFigure 2. ROBINS-I Risk of Bias Table for Nonrandomized Studies eFigure 3. Comparisons Between Data Collection Modes eTable 1. Socioeconomic Domains Used in the Included Studies eTable 2. Response Options Within Each SES Domain eTable 3. Equivalence Between Modalities of 6 Studies Reporting Equivalence Between Data Collection Modes eTable 4. Time Requirement for Each Modality eTable 5. Cost per Completed Interview for Different Modes eTable 6. Summary of Key Findings eReferences [file jamanetwopen-e2243883-s001.pdf]

Supplementary Online Content

Allen LN, Mackinnon S, Gordon I, et al. Performance and resource requirements of in-person, voice call, and automated telephone-based socioeconomic data collection modalities for community-based health programs: a systematic review. *JAMA Netw Open*. 2022;5(11):e2243883. doi:10.1001/jamanetworkopen.2022.43883

eMethods. Search Strategy

eFigure 1. RoB-2 Risk of Bias Table for Randomized Studies

eFigure 2. ROBINS-I Risk of Bias Table for Nonrandomized Studies

eFigure 3. Comparisons Between Data Collection Modes

eTable 1. Socioeconomic Domains Used in the Included Studies

eTable 2. Response Options Within Each SES Domain

eTable 3. Equivalence Between Modalities of 6 Studies Reporting Equivalence Between Data Collection Modes

eTable 4. Time Requirement for Each Modality

eTable 5. Cost per Completed Interview for Different Modes

eTable 6. Summary of Key Findings

eReferences

This supplementary material has been provided by the authors to give readers additional information about their work.

© 2022 Allen LN et al. *JAMA Network Open.*

eMethods. Search strategy

MEDLINE
Ovid MEDLINE(R) and Epub Ahead of Print, In-Process, In-Data-Review & Other Non-Indexed Citations and Daily [1946 to June 29, 2021]

1. Telephone/
2. (telephone$ or phone$).tw.
3. ((voice or phone) adj1 call$).tw.
4. (phone adj2 interview$).tw.
5. Cell Phones/
6. Smartphone/
7. (phone$ adj1 (smart or cell)).tw.
8. (smartphone$ or cellphone$).tw.
9. (mobile adj2 (phone$ or device$)).tw.
10. or/1-9
11. Text Messaging/
12. (text or texts or texting).tw.
13. MMS.tw.
14. SMS.tw.
15. short message service.tw.
16. multimedia message service.tw.
17. (automated adj2 (telephone$ or text$ or message$ or questionnaire$)).tw.
18. (telephone adj1 administered adj1 questionnaire$).tw.
19. or/11-18
20. Interviews as Topic/
21. Patient Health Questionnaire/
22. Self Report/
23. (in adj1 person$).tw.
24. (in adj1 person$ adj4 (interview$ or survey$ or question$)).tw.
25. (face adj2 face adj4 (interview$ or survey$ or question$)).tw.
26. (face-to-face adj4 (interview$ or survey$ or question$)).tw.
27. or/20-26
28. 10 and 19
29. 10 and 27
30. 19 and 27
31. 10 and 19 and 27
32. 28 or 29 or 30 or 31
33. Vulnerable populations/ or socioeconomic factors/ or poverty/ or social class/ or Healthcare Disparities/ or Health Status Disparities/ or Poverty areas/ or Urban population/
34. (equit$ or inequit$ or inequalit$ or disparit$ or equality).tw.
35. (ethnic$ or race or racial$ or caste$).tw.
36. ((social$ or socio-economic or socioeconomic or economic or structural or material) adj3 (advantage$ or disadvantage$ or exclude$ or exclusion or include$ or inclusion or status or position or gradient$ or hierarch$ or class$ or determinant$)).tw.

© 2022 Allen LN et al. *JAMA Network Open.*

37. (health adj3 (gap$ or gradient$ or hierarch$)).tw.
38. exp education/ or educational status/ or employment/ or income/ or occupations/ or social conditions/
39. (SES or SEP or sociodemographic$ or socio-demographic$ or demographic$ or income or wealth$ or poverty or affluen$).tw.
40. (educat$ adj3 (level$ or attain$ or status or well or better)).tw.
41. (occupation or unemploy$).tw.
42. (home owner$ or tenure).tw.
43. (household adj2 (income or wealth or status)).tw.
44. ((well or better or worse) adj2 off).tw.
45. or/33-43
46. Community Health Planning/
47. Community Health Services/
48. Community Health Nursing/
49. National Health Programs/
50. State Medicine/
51. Regional Health Planning/
52. Health Planning/
53. Health Plan Implementation/
54. Health Planning Guidelines/
55. Health Care Reform/
56. Health Resources/
57. Health Priorities/
58. Health Services Research/
59. "health services needs and demand"/
60. Needs Assessment/
61. State Health Plans/
62. Regional Health Planning/
63. Primary Health Care/
64. Health Services, Indigenous/
65. Rural Health Services/
66. Mobile Health Units/
67. randomized controlled trial/ or controlled clinical trials as topic/ or randomized controlled trials as topic/
68. (randomized or randomised or randomly or RCT).tw.
69. outcome assessment, health care/
70. comparative study/ or evaluation studies/ or meta-analysis/ or multicenter study/ or "systematic review"/ or validation studies/
71. epidemiologic studies/ or follow-up studies/ or longitudinal studies/ or prospective studies/ or controlled before-after studies/
72. or/46-71
73. 32 and 45 and 72
74. limit 73 to yr="1999 -Current"

© 2022 Allen LN et al. *JAMA Network Open.*

Embase

1. telephone/
2. telephone interview/
3. (telephone$ or phone$).tw.
4. ((voice or phone) adj1 call$).tw.
5. (phone adj2 interview$).tw.
6. mobile phone/
7. smartphone/
8. (phone$ adj1 (smart or cell)).tw.
9. (smartphone$ or cellphone$).tw.
10. (mobile adj2 (phone$ or device$)).tw.
11. or/1-10
12. text messaging/
13. (text or texts or texting).tw.
14. MMS.tw.
15. SMS.tw.
16. multimedia message service.tw.
17. short message service.tw.
18. (automated adj2 (telephone$ or text$ or message$ or questionnaire$)).tw.
19. (telephone adj1 administered adj1 questionnaire$).tw.
20. or/12-19
21. interview/
22. (in adj1 person$).tw.
23. (in adj1 person$ adj4 (interview$ or survey$ or question$)).tw.
24. (face adj2 face adj4 (interview$ or survey$ or question$)).tw.
25. (face-to-face adj4 (interview$ or survey$ or question$)).tw.
26. or/21-25
27. 11 and 20
28. 11 and 26
29. 20 and 26
30. 11 and 20 and 26
31. 27 or 28 or 29 or 30
32. socioeconomics/
33. poverty/
34. social status/
35. social class/
36. vulnerable population/
37. health care disparity/
38. health disparity/
39. urban population/
40. (equit$ or inequit$ or inequalit$ or disparit$ or equality).tw.
41. (ethnic$ or race or racial$ or caste$).tw.

© 2022 Allen LN et al. *JAMA Network Open.*

42. ((social$ or socio-economic or socioeconomic or economic or structural or material) adj3 (advantage$ or disadvantage$ or exclude$ or exclusion or include$ or inclusion or status or position or gradient$ or hierarch$ or class$ or determinant$)).tw.
43. (health adj3 (gap$ or gradient$ or hierarch$)).tw.
44. education/
45. educational status/
46. employment/
47. employment status/
48. unemployment/
49. household income/ or family income/ or income/
50. occupation/
51. (SES or SEP or sociodemographic$ or socio-demographic$ or demographic$ or income or wealth$ or poverty or affluen$).tw.
52. (educat$ adj3 (level$ or attain$ or status or well or better)).tw.
53. (occupation or unemploy$).tw.
54. (home owner$ or tenure).tw.
55. (household adj2 (income or wealth or status)).tw.
56. ((well or better or worse) adj2 off).tw.
57. or/32-56
58. public health/
59. health care planning/
60. community care/
61. community health nursing/
62. national health service/
63. health care policy/
64. health services research/
65. health service/
66. primary health care/
67. indigenous health care/
68. rural health care/
69. randomized controlled trial/ or controlled clinical trial/ or "randomized controlled trial (topic)"/

70. (randomized or randomised or randomly or RCT).tw.
71. outcome assessment/
72. comparative study/
73. evaluation study/
74. "systematic review"/ or "systematic review (topic)"/ or meta analysis/
75. epidemiology/
76. prospective study/
77. longitudinal study/
78. follow up/
79. or/58-78
80. 31 and 57 and 79
81. limit 80 to yr="1999 -Current"

© 2022 Allen LN et al. *JAMA Network Open.*

Global Health

1. mobile telephones/ or telephones/
2. (telephone$ or phone$).tw.
3. ((voice or phone) adj1 call$).tw.
4. (phone adj2 interview$).tw.
5. (phone$ adj1 (smart or cell)).tw.
6. (smartphone$ or cellphone$).tw.
7. (mobile adj2 (phone$ or device$)).tw.
8. or/1-7
9. (text or texts or texting).tw.
10. (MMS or SMS).tw.
11. multimedia message service.tw.
12. short message service.tw.
13. (automated adj2 (telephone$ or text$ or message$ or questionnaire$)).tw.
14. (telephone adj1 administered adj1 questionnaire$).tw.
15. or/9-14
16. interviews/
17. (in adj1 person).tw.
18. (in adj1 person adj4 (interview$ or survey$ or question$)).tw.
19. (face adj2 face adj4 (interview$ or survey$ or question$)).tw.
20. (face-to-face adj4 (interview$ or survey$ or question$)).tw.
21. or/16-20
22. 7 and 15
23. 7 and 21
24. 15 and 21
25. 7 and 15 and 21
26. 22 or 23 or 24 or 25
27. socioeconomic status/ or socioeconomics/
28. poverty/
29. exp social classes/ or caste/ or social inequalities/ or social mobility/
30. urban population/
31. (equit$ or inequit$ or inequalit$ or disparit$ or equality).tw.
32. (ethnic$ or race or racial$ or caste$).tw.
33. ((social$ or socio-economic or socioeconomic or economic or structural or material) adj3 (advantage$ or disadvantage$ or exclude$ or exclusion or include$ or inclusion or status or position or gradient$ or hierarch$ or class$ or determinant$)).tw.
34. (health adj3 (gap$ or gradient$ or hierarch$)).tw.
35. education/
36. employment/
37. occupations/
38. income/ or household income/
39. living conditions/

© 2022 Allen LN et al. *JAMA Network Open.*

40. (SES or SEP or sociodemographic$ or socio-demographic$ or demographic$ or income or wealth$ or poverty or affluen$).tw.
41. (educat$ adj3 (level$ or attain$ or status or well or better)).tw.
42. (occupation or unemploy$).tw.
43. (home owner$ or tenure).tw.
44. (household adj2 (income or wealth or status)).tw.
45. ((well or better or worse) adj2 off).tw.
46. or/27-45
47. 26 and 46
48. limit 47 to yr="1999 -Current"

ClinicalTrials.gov

Search 1
socioeconomic AND (telephone OR phone) AND (interview OR face-to-face OR in-person) AND community | Interventional Studies

Search 2
socioeconomic AND (telephone OR phone) AND (text OR SMS OR MMS) AND community | Interventional Studies

Search 3
socioeconomic AND (text OR SMS OR MMS) AND (interview OR face-to-face OR in-person) AND community | Interventional Studies

WHO ICTRP

Search 1
socioeconomic AND telephone AND interview AND community

Search 2
socioeconomic AND telephone AND text AND community

Search 3
socioeconomic AND text AND interview AND community

OpenGrey

socioeconomic AND (telephone OR phone OR text OR interview OR face-to-face OR in-person) AND community

© 2022 Allen LN et al. *JAMA Network Open.*

	Randomisation process	Deviations from intended interventions	Missing outcome data	Measurement of the outcome	Selection of the reported result	**Overall**
Ellen 2002[1]	−	+	+	+	+	+
English 2019[2]	−	+	+	−	−	−
Greenleaf 2020[3]	−	+	−	+	+	+
Nagelhout 2010[4]	−	+	−	+	+	+
Pariyo 2019[5]	+	+	+	+	+	+
Corkrey 2002[6]	×	+	−	−	−	−
Ashigbie 2021[7]	×	+	+	+	+	−

+ Low risk − Some concerns × High risk

eFigure 1: RoB-2 risk of bias table for randomized studies

© 2022 Allen LN et al. *JAMA Network Open.*

	Confounding	Selection of participants	Classification of interventions	Deviations from intended interventions	Missing data	Measurement of outcomes	Selection of reported results	**Overall**
Chittleborough 2008[8]	+	−	+	+	+	+	+	+
Gagliardi 2020[9]	−	−	+	+	+	+	+	−
Graham 2006[10]	+	+	+	+	−	+	+	+
Graham 2008[11]	+	+	+	+	+	+	−	+

+ Low risk   − Some concerns   × High risk

eFigure 2: ROBINS-I risk of bias table for non-randomised studies

© 2022 Allen LN et al. *JAMA Network Open.*

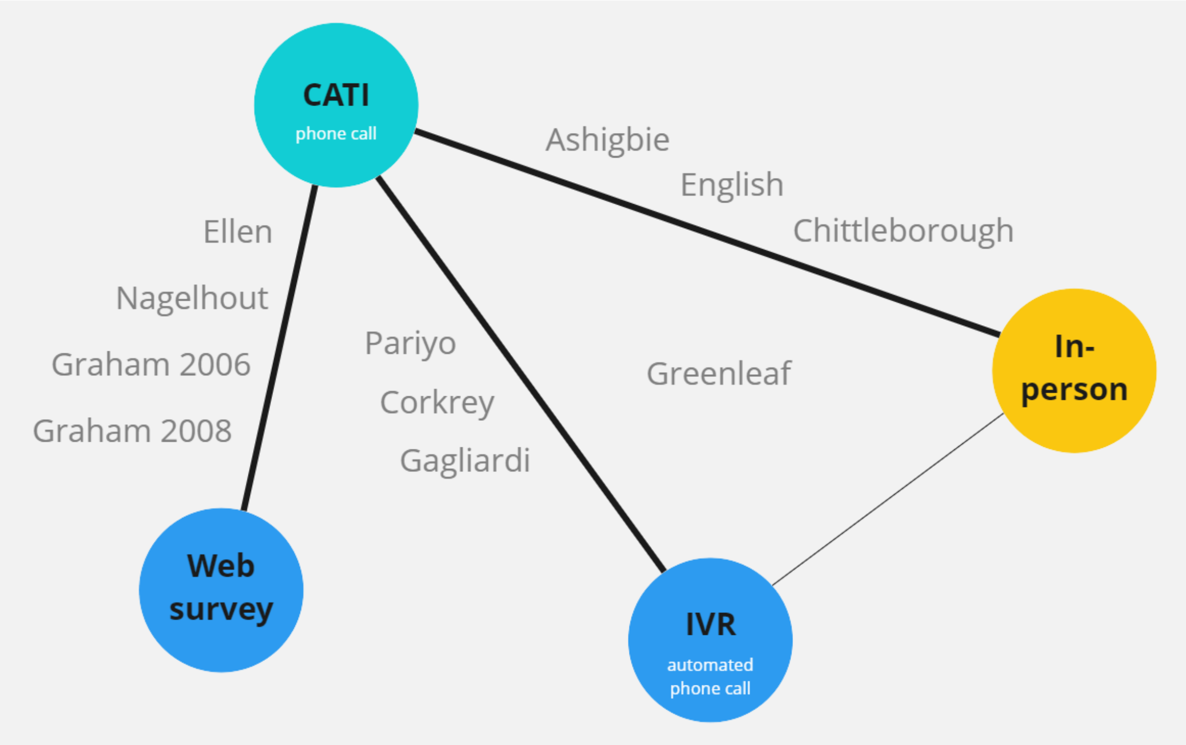

eFigure 3: Comparisons between data collection modes

Blue circles: Automated modes; Green: Voice call; Gold: in-person
Note: Line thickness represents number of studies comparing the connected modalities. Superscript numbers are references

© 2022 Allen LN et al. *JAMA Network Open.*

e Table 1: Socioeconomic domains used in the included studies

Domain	Number of studies (%)
Education	9 (82%)
- Schooling[1,3]	
- Highest level of education[2,4,5,7,8,10,11]	
Marital status	5 (45%)
- Marital status[3,4,6,8,10]	
Employment	4 (36%)
- Employment status[2,6,8,10]	
- Occupation[8]	
Household income	4 (36%)
- Household income[2,8,10,11]	
Residence	3 (27%)
- Residence[3,5,8]	
Immigrant status	2 (18%)
- Country of birth[6,8]	
Housing	1 (9%)
- Household structure (parents/guardians)[1]	1 (9%)
Healthcare Access	1 (9%)
- Primary care registration[9]	
- Health insurance[9]	
Race	1 (9%)
- Race[10]	
Wealth	1 (9%)
- Wealth quintiles[7]	
**Parent's education**	1 (9%)
- Mother's highest completed level of education[8]	
- Father's highest completed level of education[8]	

© 2022 Allen LN et al. *JAMA Network Open.*

eTable 2: Response options within each SES domain

Domain	Response options [study]	Number of studies (%)
Education		9 (82%)
- Schooling	- Enrolled in school<br>- Not enrolled in school [Ellen]<br><br>- Ever attended school<br>- Never attended school [Greenleaf]	
- Highest level of education	- Preschool/none<br>- Primary school not completed<br>- Primary school<br>- Secondary school<br>- Higher than secondary school<br>- Vocational school (post-primary)[Ashigbie]<br><br>- None<br>- School<br>- Vocational<br>- University [Corkrey]<br><br>- Still at school<br>- Left school <15y<br>- Left >15y<br>- Trade/apprenticeship<br>- Certificate/diploma<br>- Bachelor degree or higher[Chittleborough]	

© 2022 Allen LN et al. *JAMA Network Open.*

	- Non–high school graduate<br>- High school graduate<br>- College graduate [English]<br><br>- Less than a high school degree<br>- High school degree/GED<br>- 1-3 years college<br>- ≥4 years college [Graham, 2006]<br><br>- Low (primary education and lower pre-vocational secondary education)<br>- Medium (middle pre-vocational secondary) [Nagelhout]<br><br>- No school<br>- Primary<br>- Secondary<br>- University/tertiary plus [Pariyo]	
**Parent's education**		1 (9%)
- Mother’s highest completed level of education	- Completed primary school<br>- Some high school<br>- Completed high school Trade/Diploma<br>- University degree or higher<br>- Other<br>- Don’t know[Chittleborough]	
- Father’s highest completed level of education	- Completed primary school<br>- Some high school<br>- Completed high school Trade/Diploma	

© 2022 Allen LN et al. *JAMA Network Open.*

	- University degree or higher<br>- Other<br>- Don't know[Chittleborough]	
Marital status		5 (45%)
- Marital status	- Married<br>- Divorced/separated<br>- Widowed<br>- Never married [Corkrey]<br><br>- Married/de facto<br>- Separated/divorced/widowed<br>- Never married [Chittleborough]<br><br>- Currently in union<br>- Currently not in union [Greenleaf]<br><br>- Not married<br>- Not married but living together<br>- Married<br>- Registered partners<br>- Widowed<br>- Divorced<br>- Refused to answer [Nagelhout]<br><br>- Response options not specified [Graham 2006]	
Employment		4 (36%)

© 2022 Allen LN et al. *JAMA Network Open.*

- Employment status	- In labour force<br>- Not in labour force [Corkrey]<br><br>- Full time<br>- Part time/casual<br>- Unemployed/unable to work<br>- Home duties<br>- Retired<br>- Student<br>- Not stated [Chittleborough]<br><br>- Employed<br>- Unemployed<br>- Other [English]<br><br>- Response options not specified [Graham 2006]	
- Occupation	- Australian Standard Classification of Occupations (ASCO)<br>- 1) Manager, administrator, professional,<br>- 2)Associate professional<br>- 3) Tradesperson, advanced clerical or service,<br>- 4) Intermediate clerical/ service/ sales/ production/ transport<br>- 5) Elementary clerical/sales/service/ labourer.<br>- Other: home duties, student, never worked or not stated [Chittleborough]	

© 2022 Allen LN et al. *JAMA Network Open.*

Household income		4 (36%)
- Household income	- <$20k<br>- $20-40k<br>- $40-60k<br>- >$60k [Chittleborough]<br><br>- <$10 000<br>- $10 000 to $19 999<br>- $20 000 to $34 999<br>- $35 000 to $49 999<br>- ≥$50 000 [English]<br><br>- <$10k<br>- $10-20k<br>- $20-30k<br>- $30-40k<br>- $40-50k<br>- $50k-$75<br>- $75-100k<br>- >$100 [Graham, 2006 & Graham, 2008]	
Residence		3 (27%)
- Residence	- Metropolitan<br>- Country [Chittleborough]<br><br>- Urban<br>- Rural [Greenleaf]	

© 2022 Allen LN et al. *JAMA Network Open.*

	- Urban<br>- Rural [Pariyo]	
Immigrant status		2 (18%)
- Country of birth	- Australia<br>- Other [Corkrey]<br><br>- Australia<br>- UK/Ireland<br>- Other [Chittleborough]	
Housing		1 (9%)
- Household structure	- One parent<br>- Two parent<br>- Other parent/guardian structure [Ellen]	1 (9%)
Healthcare Access		1 (9%)
- Primary care registration	- Still enrolled in the practice<br>- No longer enrolled in the practice [Gagliardi]	
- Health insurance	- Commercial<br>- Medicaid<br>- Medicare<br>- Other<br>- Missing [Gagliardi]	
Race	- White<br>- Black<br>- Asian<br>- American Indian	1 (9%)

© 2022 Allen LN et al. *JAMA Network Open.*

	- Alaskan Native [Graham]	
Wealth	- Wealth Quintiles 1-5 [Ashigbie]	1 (9%)

© 2022 Allen LN et al. *JAMA Network Open.*

eTable 3: Equivalence between modalities of 6 studies reporting equivalence between data collection modes

Study	SES Domain[a]	Modalities	Cohen's K (Standard error)
Pariyo[5]	Education Bangladesh	CATI then IVR	0.51 (SE: 0.04)
		IVR then CATI	0.60 (SE: 0.03)
	Education Tanzania	CATI then IVR	0.67 (SE: 0.06)
		IVR then CATI	0.03 (SE 0.01)
Greenleaf[3]	Ever attended school (yes/no) Burkina Faso	F2F v IVR	0.68 (SE not provided)
		F2F v CATI	0.84 (SE not provided)
Pariyo[5]	Urban/rural Bangladesh	CATI then IVR	0.72 (SE: 0.07)
		IVR then CATI	0.75 (SE: 0.05)
	Urban/rural Tanzania	CATI then IVR	0.53 (SE: 0.07)
		IVR then CATI	0.57 (SE: 0.05)
Greenleaf[3]	Marital status Burkina Faso	F2F v IVR	0.66 (SE not provided)
		F2F v CATI	0.70 (SE not provided)
Graham 2006[10]	Household income USA	CATI v web	0.93 (95CI: 0.85-1.00)
Graham 2008[11]		CATI v web-survey	>0.84[b] (SE not provided)

Abbreviations: F2F, face to face; SES, Socioeconomic status; CATI, computer-assisted telephone interview; IVR, interactive voice response, SE, standard error

[a]*The denominator for each domain is the entire population for each study listed in Table 1*

[b]Graham 2008 presented equivalence data on household income broken down by ethnic group and income status: for racial/ethnic minority groups K=0.87 and for non-Hispanic whites K=0.94. For low-income groups K = 0.84 and for high-income K= 0.92

eTable 4: Time requirement for each modality

Study	Domains	CATI	Comparator	Ratio CATI/Comparator
Ashigbie[7]	Education and wealth	12.8 mins	8.3 mins[a] In-person	1.48
Nagelhout[4]	Education and marital status	40 mins	Not reported Web-survey	N/A
English[2]	Education, income, and employment status	Not reported	45 mins In-person	N/A

Abbreviations: CATI, computer-assisted telephone interview
[a]Does not include travel time

© 2022 Allen LN et al. *JAMA Network Open.*

eTable 5: Cost per completed interview for different modes

Study	CATI	Comparator	Ratio CATI/ Comparator	Costs included[a]
Ellen 2002[1]	US$58.51	US$99.38 Web-survey	0.59	'We computed the costs per completed case for each mode by combining actual costs for interviewers, mailing, telephones, travel, incentives, and supplies.'
Nagelhout 2010[4]	US$90	US$22 Web-survey	4.09	'Fieldwork costs and reimbursements' Web survey respondents 'received compensation for their participation in the survey by earning points for every answered question, as is standard procedure in the TNS NIPObase web panel. The points could be exchanged into money, which ranged between 5 and 7 Euro for this survey' 'Each telephone respondent received a reimbursement of 10 euro by mail after completing the survey.'
Corkrey 2002[6]	AU$6.03	AU$7.92 IVR	0.76	'Costs were calculated by summing salary cost and accumulated telephone call charges. Salary cost was calculated by multiplying the total of interview duration, including time spent waiting on hold in Hybrid I, and noncontact attempt duration by the interviewer's salary rate.'
Greenleaf 2020[3]	US$51.67	US$143.55 IVR	0.36	'Survey implementation costs were compared as total cost per mode and cost per completed interview. Itemized costs were summarized by category of cost (procurement, preparatory and pilot, training, and data collection) and by mode. Many itemized costs were equivalent between the two modes. Assumptions were made to adjust the duration of training and data collection for each mode as if they had been conducted as stand-alone activities in order to determine a more accurate cost per mode. Specifically, we assumed each of the two modes used four supervisors and 15 interviewers, training was three days for supervisors and four days for interviewers, and that it would take 10 days to call all

© 2022 Allen LN et al. *JAMA Network Open.*

				1,766 women, including up to six call- backs per mode. Most costs were incurred in local currency, the West African CFA franc. Costs in CFA were converted to the US dollar (USD) using the 2017 average exchange rate of 1 USD: 582 CFA. We did not include the cost of software development in the fieldwork cost comparisons. As a joint software solution was created to meet the needs for the study for both CATI and Hybrid IVR and therefore the costs for software specific for each mode were not estimated, the assumption was made that the cost of building the software was the same for both modes.'
Gagliardi 2020[9]	US$31.32	US$13.68 IVR	2.29	'We also measured panel management outreach costs, consisting of the personnel costs for the panel managers and the fee charged by CipherHealth for a 1-year service agreement. Costs did not include the time spent by University of California San Francisco Health and CipherHealth personnel in designing the Automated Call systems.'
English[b] 2019[2]	US$211	US$192 In-person	1.10	'Total costs for the telephone survey administration were $35 000, equivalent to the value of the contract with the state Behavioral Risk Factor Surveillance System (BRFSS) survey unit. Costs for the in-person survey administration included AASTEC labor (epidemiologists, community interviewers, and data entry technicians), participant incentives, and interviewer mileage reimbursement.'
Ashigbie 2021[7]	US$16.86	US$38.84 In-person	0.43	'Bottom-up itemisation of all major cost components including air time, transport & personnel'

Abbreviations: CATI, computer-assisted telephone interviews*; IVR, interactive voice response*

[a] *All costs presented are for completion of the entire survey, including transport and personnel costs. Corkrey also gathered data on cost per completed interview that excluded costs associated with failed attempts: CATI: AU$4.97 and IVR AU$2.27 per interview.*

[b] *The phone survey was shorter than the F2F in the English et al study.*

eTable 6: Summary of key findings

Outcomes	Key findings	Certainty of evidence
**Response rate**	CATI is equivalent to in-person survey, and both achieve very high levels of response[8]	Low
	CATI is equivalent to web-survey and both achieve very high levels of response[4]	Very low
	CATI is superior to IVR[3]	Moderate
**Equivalence**	Moderate to almost perfect agreement between IVR, CATRI, web-survey, and F2F approaches for education, location, marital status, and income domains[5,10–12]	Moderate
**Time requirements**	CATI surveys take 1.5 times longer to complete than in-person surveys, excluding travel time[7]	Very low
**Costs**	Automated and remote modes may be more cost-effective than in-person and synchronous modes when response rates are high and translation costs are low[1–4,6,7,9]	Very low
**Acceptability**	Responders feel that CATI, web-survey, and IVR are highly acceptable methods for eliciting SES data[1,6]	Moderate

Abbreviations: CATI, computer assisted telephone interview; IVR, Interactive voice response;F2F, Face to face

© 2022 Allen LN et al. *JAMA Network Open.*

eReferences

1. Ellen JM, Gurvey JE, Pasch L, Tschann J, Nanda JP, Catania J. A randomized comparison of A-CASI and phone interviews to assess STD/HIV-related risk behaviors in teens. Journal of Adolescent Health. 2002 Jul;31(1):26–30.

2. English KC, Espinoza J, Pete D, Tjemsland A. A Comparative Analysis of Telephone and In-Person Survey Administration for Public Health Surveillance in Rural American Indian Communities. J Public Health Manag Pract. 2019 Oct;25 Suppl 5, Tribal Epidemiology Centers: Advancing Public Health in Indian Country for Over 20 Years:S70–6.

3. Greenleaf AR, Gadiaga A, Choi Y, Guiella G, Turke S, Battle N, et al. Automated and Interviewer-Administered Mobile Phone Surveys in Burkina Faso: Sociodemographic Differences Among Female Mobile Phone Survey Respondents and Nonrespondents. JMIR Mhealth Uhealth. 2020 Jul 14;8(7):e17891.

4. Nagelhout GE, Willemsen MC, Thompson ME, Fong GT, van den Putte B, de Vries H. Is web interviewing a good alternative to telephone interviewing? Findings from the International Tobacco Control (ITC) Netherlands Survey. BMC Public Health. 2010 Jun 18;10:351.

5. Pariyo GW, Greenleaf AR, Gibson DG, Ali J, Selig H, Labrique AB, et al. Does mobile phone survey method matter? Reliability of computer-assisted telephone interviews and interactive voice response non-communicable diseases risk factor surveys in low and middle income countries. Maulik PK, editor. PLoS ONE. 2019 Apr 10;14(4):e0214450.

6. Corkrey R, Parkinson L. A comparison of four computer-based telephone interviewing methods: Getting answers to sensitive questions. Behavior Research Methods, Instruments, & Computers. 2002 Aug 1;34(3):354–63.

7. Ashigbie PG, Rockers PC, Laing RO, Cabral HJ, Onyango MA, Mboya J, et al. Phone-based monitoring to evaluate health policy and program implementation in Kenya. Health Policy and Planning. 2021 May 1;36(4):444–53.

8. Chittleborough CR, Taylor AW, Baum FE, Hiller JE. Non-response to a life course socioeconomic position indicator in surveillance: comparison of telephone and face-to-face modes. BMC Med Res Methodol. 2008 Dec;8(1):54.

9. Gagliardi KS, Coleman S, Intinarelli G, Karliner L, Appelle N, Taylor B, et al. An Automated Telephone Call System Improves the Reach and Cost-effectiveness of Panel Management Outreach for Cancer Screening. Journal of Ambulatory Care Management. 2020 Apr;43(2):148–56.

10. Graham AL, Papandonatos GD, Bock BC, Cobb NK, Baskin-Sommers A, Niaura R, et al. Internet- vs. telephone-administered questionnaires in a randomized trial of smoking cessation. Nicotine Tob Res. 2006 Dec;8(Suppl 1):S49–57.

11. Graham AL, Papandonatos GD. Reliability of Internet- Versus Telephone-Administered Questionnaires in a Diverse Sample of Smokers. J Med Internet Res. 2008 Mar 26;10(1):e8.

12. Greenleaf AR, Gibson DG, Khattar C, Labrique AB, Pariyo GW. Building the Evidence Base for Remote Data Collection in Low- and Middle-Income Countries: Comparing Reliability and Accuracy Across Survey Modalities. J Med Internet Res. 2017 May 5;19(5):e140.

© 2022 Allen LN et al. *JAMA Network Open.*
